# Compression of the lateral antebrachial cutaneous nerve by a traumatic arteriovenous fistula

**DOI:** 10.1515/tnsci-2020-0102

**Published:** 2020-04-20

**Authors:** Min Cheol Chang, Mathieu Boudier-Revéret, Ming-Yen Hsiao

**Affiliations:** Department of Physical Medicine and Rehabilitation, College of Medicine, Yeungnam University, Namku, Taegu, Republic of Korea; Department of Physical Medicine and Rehabilitation, University of Montreal Health Center, Montreal, Canada; Department of Physical Medicine and Rehabilitation, National Taiwan University Hospital, College of Medicine, National Taiwan University, Taipei, Taiwan

To the editor

A 50-year-old man presented to the hospital with a severe lancinating pain in the left lateral forearm. One month prior to his visit, the patient had been attacked by a flock of birds, at which time he had sustained a laceration on the lateral side of the left proximal forearm. He complained of persisting severe lancinating pain on the lateral side of his left forearm even after complete healing of wound. No palpable mass was found. There was no motor weakness on examination; however, there were hypoesthesia and allodynia over the left lateral forearm, with provoked paresthesia by palpation of the scar. Because the lancinating forearm pain arose after the patient sustained a laceration in a bird attack, an ultrasound (US) examination (13–18 MHz linear probe) was performed to evaluate the area around the wound.

**Figure 1:**
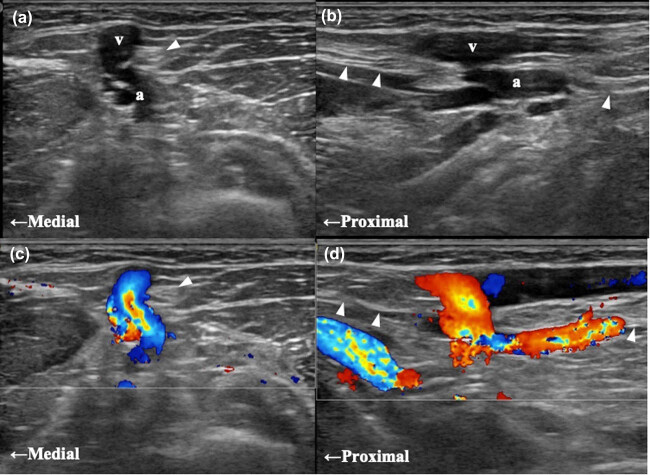
Images from the ultrasonography examination at the level of the proximal forearm. Grayscale US images on transverse (a) and longitudinal axes (b) reveal that the cephalic vein (v) was connected to the radial artery (a), and the two vessels were focally dilated. Color Doppler images on transverse (c) and longitudinal axes (d) reveal arterial blood flow at the junction between the two vessels and inside the cephalic vein. These findings are concordant with an AVF. The LACN (arrow heads) was encased by the two vessels and the AVF.

Just below the laceration wound, grayscale US images on longitudinal and transverse axes revealed a connection between the radial artery and cephalic vein and focal dilatations of those two vessels at the connected areas (Figure 1a and b). The color Doppler images revealed arterial blood flow at the junction between the radial artery and the cephalic vein and inside the cephalic vein (Figure 1c and d). These findings were consistent with an arteriovenous fistula (AVF) between the radial artery and cephalic vein. Furthermore, the US revealed that the left lateral antebrachial cutaneous nerve (LACN) was encased by the radial artery, cephalic vein, and AVF (Figure 1). We found focal swelling and hypoechoic changes of the nerve proximal to the encased segment. The US tracking of the left LACN showed no significant abnormal findings other than the focal swelling and hypoechoic change in that region. The patient’s pain was located in the cutaneous region innervated by the left LACN. Considering the aforementioned findings, we concluded that the compression of the left LACN caused by the traumatic AVF was responsible for the persisting pain.

LACN is the terminal cutaneous sensory branch of the musculocutaneous nerve. It is most commonly injured during venipunctures as it lies in close proximity to the cephalic vein, and it can also be involved in distal biceps brachii tendon pathology, as it lies just lateral to the tendon at the cubital fossa level [1]. In clinical practice, cutaneous nerve disorders tend to be overlooked by clinicians even though cutaneous nerves are susceptible to external compression and iatrogenic injuries due to their superficial course. A nerve conduction study (NCS) is usually used for evaluation; however, NCS assessment of cutaneous nerve pathology is sometimes challenging due to the anatomical variations in distribution and inadequate sensitivity of detecting minor lesions. In addition, NCS is limited in that it cannot visualize the morphological changes in the cutaneous nerves and investigate the structures surrounding them. These limitations are in part responsible for the underdiagnosis of cutaneous nerve disorders by clinicians.

US can aid in visualizing nerve morphology and detecting nerve pathology and potentially the underlying cause. US can also help guiding perineural steroid injection, nerve hydrodissection, radiofrequency ablation, and can help plan a surgical release procedure more accurately [2]. Therefore, US has been expected to overcome the limitations of NCS in the diagnosis of cutaneous nerve disorders. Recently, with efforts made by clinicians and researchers, new knowledge has accumulated on methods for tracking several cutaneous nerves using US [1,3]. Additionally, the development of high-resolution US allows clinicians to evaluate cutaneous nerve pathologies in greater detail. In 2019, Wu and Boudier-Reveret demonstrated that US is useful to diagnose pathology of the cutaneous nerve by identifying an entrapment of the LACN within a post-surgical scar [4]. This study is the first to describe an entrapment of the LACN caused by an AVF.

AVF between the radial artery and the cephalic vein has already been described as a result of penetrating injury [5], although rare, and even more rarely, without associated penetrating trauma [6]. However, they are commonly used vessels to surgically make a hemodialysis fistula [7]. To the best of our knowledge, only one case of nerve compression by an AVF has been reported. After insertion of a central monitoring catheter in the jugular vein, an AVF had developed between the vertebral artery and jugular vein, which compressed the brachial plexus and caused motor weakness [8].

In this study, by reporting a case of LACN compression by a traumatic AVF diagnosed using US, we emphasized the importance of evaluating cutaneous nerves, particularly in patients presenting with neuropathic pain in the distribution of cutaneous nerves.
